# UHPLC-MS Chemical Fingerprinting and Antioxidant, Antiproliferative, and Enzyme Inhibition Potential of *Gaultheria pumila* Berries

**DOI:** 10.3390/metabo11080523

**Published:** 2021-08-07

**Authors:** Carlos Fernández-Galleguillos, Luisa Quesada-Romero, Adrián Puerta, José M. Padrón, Ernane Souza, Javier Romero-Parra, Mario J. Simirgiotis

**Affiliations:** 1 Instituto de Farmacia, Facultad de Ciencias, Universidad Austral de Chile, Campus Isla Teja, Valdivia 5090000, Chile; carlos.fernandez@uach.cl; 2 Facultad de Ciencias Para el Cuidado de la Salud, Universidad San Sebastián, Valdivia 5090000, Chile; 3 BioLab, Instituto Universitario de Bio-Orgánica Antonio González (IUBO-AG), Universidad de La Laguna, 38206 La Laguna, Spain; apuertaa@ull.es (A.P.); jmpadron@ull.es (J.M.P.); 4 The Lloyd L. Gregory School of Pharmacy, Palm Beach Atlantic University, West Palm Beach, FL 33401, USA; ernane.souza@pba.edu; 5 Departamento de Química Orgánica y Fisicoquímica, Facultad de Ciencias Químicas y Farmacéuticas, Universidad de Chile, Olivos 1007, Casilla 233, Santiago 6640022, Chile; javier.romero@ciq.uchile.cl; 6 Center for Interdisciplinary Studies on the Nervous System (CISNe), Universidad Austral de Chile, Valdivia 5090000, Chile

**Keywords:** gaultheria, phenolics, enzyme inhibition, native berries, antioxidant

## Abstract

*Gaultheria pumila* (Ericaceae) (known as Chaura or Mutilla) is a Chilean native small shrub that produces berry fruits consumed by local Mapuche people. In this study, the chemical fingerprinting and antioxidant, enzyme inhibition, and antiproliferative activities of the berries were investigated for the first time. Thirty-six metabolites were identified in the fruits by ultra-high performance liquid chromatography-photodiode array detection, hyphenated with Orbitrap mass spectrometry analysis (UHPLC-DAD-Orbitrap-MS). Metabolites, included anthocyanins, phenolic acids, flavonoids, iridoids, diterpenes, and fatty acids. Moderate inhibitory activities against acetylcholinesterase (7.7 ± 0.3 µg/mL), butyrylcholinesterase (34.5 ± 0.5 µg/mL), and tyrosinase (3.3 ± 0.2 µg/mL) enzymes were found. Moreover, selected major compounds were subjected to docking assays in light of their experimental inhibition. Results indicated that hydrogen bonding, π–π interaction, and a salt bridge interaction contributed significantly. *Gaultheria pumila* berries showed a total phenolic content of 189.2 ± 0.2 mg of gallic acid equivalents/g, total flavonoid content of 51.8 ± 0.1 mg quercetin equivalents/g, and total anthocyanin content of 47.3 ± 0.2 mg of cianydin-3-glucoside equivalents/g. Antioxidant activity was assessed using DPPH (92.8 ± 0.1 µg/mL), FRAP (134.1 ± 0.1 μmol Trolox equivalents/g), and ORAC (4251.6 ± 16.9 μmol Trolox equivalents/g) assays. Conversely, *Gaultheria pumila* showed a scarce antiproliferative potential against several solid human cancer cells. Our findings suggest that *Gaultheria pumila* berries have several bioactive metabolites with inhibitory effects against acetylcholinesterase, butyrylcholinesterase, and tyrosinase, and have the potential for use in food supplements.

## 1. Introduction

The human consumption of native berries has been increasing worldwide in part due to their potential impact on health promotion and disease prevention. Fruit berries from a variety of species have been associated with a reduction in the risk of cardiovascular, neurodegenerative, and inflammatory diseases, as well as potential benefits against some forms of cancer [1]. The effects against reactive oxygen species (ROS) have been described as the main antioxidant protective mechanism [2]. Chile possesses a high biodiversity of native berries, which are mainly found in the central-southern region of the country and include different genuses such as *Aristotelia*, *Fragaria*, *Berberis*, *Ugni*, and *Gaultheria*, among others. The phytochemical and biological activities of plants belonging to those genuses have been reported previously [3].

The *Gaultheria* genus (Ericaceae) includes approximately 135 species, many of which have been used in traditional medicine to treat several diseases [4]. Some of their chemical constituents and pharmacological properties have been extensively investigated [5]. Antioxidant, antibacterial, anti-inflammatory, anticancer, and enzyme inhibitory properties have been reported in the scientific literature [6,7,8,9]. In Chile, the genus *Gaultheria* is distributed from the central region (Región Metropolitana) to the extreme south (Región de Magallanes), and twelve species have been formally registered including *G. angustifolia*, *G. antarctica*, *G. cespitosa*, *G. insana*, *G. mucronata*, *G. nubigena*, *G. phyllireifolia*, *G. poeppigii*, *G. tenuifolia*, *G. renjifoana*, *G. racemulosa*, *and G. pumila* [10]. Some of these plants are edible species (*G. tenuifolia*, *G. phillyreifolia*, *G. poeppigii*, *G. mucronata*, and *G. pumila*).

Anthocyanins and iridoids with important antioxidant activities have been isolated from the native berries of *G. phillyreifolia* and *G. poeppigii* [11]. The quantitative and qualitative profiles of phenolic compounds from different colored genotypes of *G. poeppigii* berries and their antioxidants activities have been evaluated [12]. Anthocyanins, proanthocyanidins, phenylpropanoids, iridoids, and flavonols have been isolated from *G. tenuifolia* berries [13]. The anthocyanin profiles, total anthocyanin content, and antioxidant activities from *G. mucronata* and *G. antarctica* have also been described [14], as well as the flavonols and hydroxycinnamic acid profiles [15]. In addition, morphometric analysis and the chemical characterization (total anthocyanin content and pectin) of red, pink, and white *G. pumila* berries have been investigated [16].

**Figure 1:**
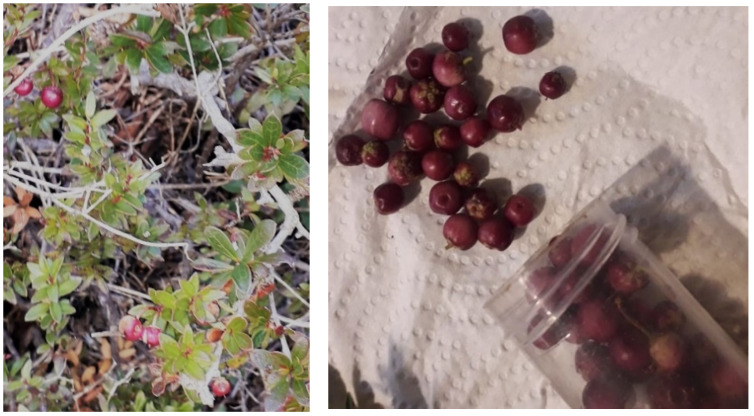
*Gaultheria pumila* (Ericaceae) shrub in Parque Nacional Conguillío (**left**) and *Gaultheria pumila* berries (**right**).

*Gaultheria pumila* is a native small bush (2–3 cm tall) that produces a tasty, aromatic, and flavored fruit popularly known as “Chaura” or “Mutilla” and is consumed by local Mapuche communities (Figure 1).

To date, no chemical fingerprinting of the metabolites from *G. pumila* berries have been investigated. To the best of our knowledge, there are no scientific reports concerning to the antiproliferative and enzyme inhibition potential. Ultra-high-resolution chromatography (UHPLC) coupled to mass spectrometry (MS) is a fast and modern technique that serves to provide information and compare the chemical profile of different metabolites that are naturally produced by plants [17,18]. Our group recently investigated the fingerprinting of Chilean native plants as well as their antioxidant and enzyme inhibitory properties [19,20]. In the present work, we report for the first time the chemical fingerprinting of *G. pumila* berries by UHPLC-MS analysis and the enzyme inhibition (against acetylcholinesterase, butyrylcholinesterase, and tyrosinase), as well as the antioxidant and antiproliferative activities.

## 2. Materials and Methods 

### 2.1. Chemicals

Ultra-pure water (<5 µg/L TOC) was obtained from the water purification systems Arium 126 61316-RO, in addition to an Arium 611 UV unit (Sartorius, Goettingen, Germany). Methanol (HPLC grade) and formic acid (MS grade) were purchased from J. T. Baker (Phillipsburg, NJ, USA). Commercial Folin–Ciocalteu (FC) reagent, 2,2-diphenyl-1-picrylhydrazyl (DPPH), ferric chloride hexahydrate, 2,4,6-tris(2-pyridyl)-s-triazine, trolox, quercetin, gallic acid, Amberlite^®^ resin (XAD4), dimethyl sulfoxide (DMSO), acetylcholinesterase (AChE), butyrylcholinesterase (BChE), phosphate buffer, LDOPA, tyrosinase, kojic acid, trichloroacetic acid (Merck, Darmstadt, Germany), fetal calf serum (FCS, Gibco, Grand Island, NY, USA), L-glutamine (Merck, Darmstadt, Germany), penicillin G (Sigma, St. Louis, MO, USA), streptomycin (Sigma, St. Louis, MO, USA), sulforhodamine B (SRB, Sigma, St. Louis, MO, USA), and HPLC standards (cyandin-3-*O*-glucoside, delphinidin-3-*O*-galactoside, cyanidin-3-*O*-galactoside, cyanidin-3-*O*-arabinoside, and quercetin-3-*O*-arabinoside) with purity higher than 95% by HPLC were purchased from Sigma Aldrich Chem. Co. (St, Louis, MO, USA) or Extrasynthèse (Genay, France).

### 2.2. Plant Material

*G. pumila* (one sample, ripe fruits, purple morphotype, and sweet flavor) was collected by hand from the National Park Conguillío (Mirador de los Cóndores), Región de La Araucanía, Chile, in October 2019 (38°43′16.0″ S, 71°39′41.5″ W). The sample was authenticated by the botanist Jorge Macaya from the University of Chile, Santiago, Chile. The sample was washed with distilled water and kept in an ultra-freezer at a desired temperature (−95 °C). A voucher specimen (voucher number GP-102019) was deposited in the Laboratory of Natural Products of the Universidad Austral de Chile (Chile).

### 2.3. Extraction Procedures

*G. pumila* (10 g) fruits were grounded using an electric processor (Ursus Trotter, UT-PETRUS320) and extracted three times with a 100 mL mixture of MeOH:formic acid (98:2, *v*/*v*) using an ultrasonic water bath (UC-60A Biobase, Guanzhou, China) for 30 min in the dark. The resulting extract was then filtered and evaporated under reduced pressure at 36 °C to obtain 998 mg of a gummy residue extract. A column filled with Amberlite^®^ resin (XAD4, 200 g) was used to achieve the flavonoid enriched extract. The extract was suspended in water (20 mL), added to the column (200 g), and washed several times with deionized water (100 mL, 3 times); then, the compounds were desorbed with MeOH:formic acid (98:2, *v*/*v*, 100 mL) and the solution was evaporated under reduced pressure at 36 °C to give 479.23 mg (4.79%). This procedure was previously used for other edible berries [21].

### 2.4. UHPLC–DAD–MS Instrument

A UHPLC-high-resolution MS machine (Thermo Dionex Ultimate 3000 system with DAD detector controlled by Chromeleon 7.2 software hyphenated with a Thermo Q-Exactive MS focus) was used to analyze the phenolic compounds in the extract. For the analysis, 5 mg of the enriched extract were dissolved in 2 mL of methanol, filtered through a 200-µm polytetrafluoroethylene (PTFE) filter, and 10 µL were injected into the instrument [22].

### 2.5. LC Parameters and MS Parameters

Liquid chromatography was performed using a UHPLC C18 column (Acclaim, 150 × 4.6 mm ID, 2.5 µm; Thermo Fisher Scientific, Bremen, Germany) operated at 25 °C. The detection wavelengths were 280, 254, 330, and 354 nm, and photodiode array detectors were set from 200 nm to 800 nm. Mobile phases were 1% formic aqueous solution (A) and acetonitrile 1% formic acid (B). The gradient program started at 5% B at time zero; was maintained at 5% B for 5 min; went to 30% B for 10 min; was maintained at 30% B for 15 min; went to 70% B for 5 min; was maintained at 70% B for 10 min; and was finally returned to the initial conditions within 10 min and 12 min for column equilibration prior to each injection. The flow rate was 1.00 mL/min and the injection volume was 10 µL. The standards and lyophilized decoction dissolved in methanol were kept at 10 °C during its storage in the autosampler. The HESI II and Orbitrap spectrometer parameters were optimized as previously reported [23]. Briefly, the parameters are as follows: sheath gas flow rate, 75 units; auxiliary gas unit flow rate, 20; capillary temperature, 400 °C; auxiliary gas heater temperature, 500 °C; spray voltage, 2500 V (for ESI−); and S lens, RF level 30. Full scan data in positive and negative modes were acquired at a resolving power of 70,000 FWHM at *m*/*z* 200. Themass scan range was between of 100–1000 *m*/*z*; automatic gain control (AGC) was set at 3 × 10^6^ and the injection time was set to 200 ms. The chromatographic system was coupled to MS with a source II heated electro-nebulization ionization probe (HESI II). The nitrogen gas carrier (purity > 99.999%) was obtained from a Genius NM32LA (Peak Scientific, Billerica, MA, USA) generator and used as a collision and damping gas. Mass calibration for Orbitrap was performed once a day in both negative and positive modes to ensure working mass 5 ppm of accuracy. For the positive mode, a mixture of caffeine (1 mg/mL, 20 µL) and N-butylamine (1 mg/mL, 100 µL) was used, while a mixture of sodium dodecyl sulfate (1 mg/mL, 100 µL) and taurocholic acid sodium salt (1 mg/mL, 100 µL) (Sigma-Aldrich, Darmstadt, Germany) was used for the negative mode. In addition, Ultramark 1621 (Alpha Aezar, Stevensville, MI, USA) was used as the reference compound (1 mg/mL, 100 µL). These compounds were dissolved in a mixture of acetic acid (100 µL), acetonitrile (5 mL), water: methanol (1:1) (5 mL) (Merck, Santiago, Chile), and 20 μL of the mixture were infused using a Chemyx Fusion 100 μL syringe pump (Thermo Fisher Scientific, Bremen, Germany. The tentative identification of the metabolites was carried out using the analysis of the full scan mass spectra, retention index, base peaks chromatograms, fragmentation pattern, and database of MassBank of North America (MoNA). In addition, representative compounds were identified using the commercial standards presented in Section 3.1. 

### 2.6. Total Phenolic (TP), Total Flavonoid (TF), and Total Anthocyanins (TA) Content

The total phenolic (TP) and total flavonoid (TF) content of *G. pumila* berries was measured using the Folin–Ciocalteu and the AlCl_3_ method employing a Synergy HTX microplate reader (Biotek, Winoosky, VT, USA) as reported previously [24,25]. Results for TP are expressed as the mg gallic acid equivalent per g of dry plant and the results for TF are expressed as the mg quercetin equivalent per g of dry plant. Total anthocyanin (TA) content was carried out by the pH differential method [26]. Buffers at pH 1.0 and 4.5 were used and the absorbance was detected at 510 nm and 700 nm, respectively. Results were expressed as the mg cyanidin-3-glucoside equivalents/g extract [26]. The experiments were performed in triplicates and the values are reported as the mean ± SD.

### 2.7. Antioxidant Activity

#### 2.7.1. DPPH Scavenging Activity

Using a Synergy HTX microplate reader, the potential bleaching of the radical DPPH was determined using 150 µL of DPPH solution (0.075 mM) and 50 µL of extract or the standard gallic acid (curve from 10–250 μg/mL). The determinations were made in triplicates by monitoring the disappearance of DPPH at 515 nm after 30 min of reaction [22,23]. The results are expressed as IC_50_ in µg of the extract or standard per mL. The values are reported as the mean ± SD.

#### 2.7.2. Ferric-Reducing Antioxidant Power Assay (FRAP)

The FRAP assay was performed as previously described [19]. Quantification was performed using a standard curve of the antioxidant Trolox. Then, the measurement was performed using a volume of 10 µL of Trolox and 290 µL of extract in a well of the microplate and absorbance was measured at 593 nm after 5 min. Trolox was used as the standard (curve made from 1–100 µg/mL). The results were expressed in μmol of the Trolox equivalent per g of dry fruit. The experiments were performed in triplicates and the values are expressed as the mean ± SD.

#### 2.7.3. Oxygen Radical Absorbance Capacity (ORAC) Assay

The ORAC assay was performed as previously described [27]. Quantification was performed using a standard curve of the antioxidant Trolox (curve from 10–100 µM). The results were obtained by the quadratic regression equation (Trolox/samples vs. fluorescence decay curves) and expressed in μmol of the Trolox equivalents per g of dry plant. The experiments were performed in triplicates and the values are reported as the mean ± SD.

### 2.8. Determination of Cholinesterase Inhibition

Inhibition of AChE activity was determined according to the Ellman method as previously reported [23]. Briefly, DTNB was dissolved in buffer Tris-HCl at pH 8.0 containing 0.1 M NaCl and 0.02 M MgCl_2_. Then, a filtered sample solution dissolved in deionized water (50 µL, the final concentration of the plate was ranging from 0.05 to 25 μg/mL) was mixed with 125 µL of 5-dithio-bis (2-nitrobenzoic) acid (DTNB), acetylcholinesterase (AChE), or butyrylcholinesterase (BuChE) solution (25 µL) dissolved in Tris-HCl buffer at pH 8.0 in a 96-well microplate and was incubated for 15 min at 25 °C. Initiation of the reaction was performed by the addition of acetyl-thiocholine iodide (ATCI) or butyryl-thiocholine chloride (BTCl) (25 µL). In addition, a blank was prepared by adding the solution sample to all reagents without the enzyme(s) (AChE or BuChE) solutions. The sample and blank absorbances were then read at 405 nm after 10 min of incubation at 25 °C. The absorbance of the sample was subtracted from that of the blank and the cholinesterase inhibitory capacity was expressed as IC_50_ (µg/mL). A Synergy HTX Multi-Mode Microplate Reader (BioTek Instruments Inc., Foster City, CA, USA) was used. Three experiments were performed in triplicates in each case and the values are reported as the mean ± SD. Galanthamine was used as positive control.

### 2.9. Tyrosinase Inhibition Assay

Tyrosinase activity was assessed using the dopachrome method [28]. G. pumila berries (20 µL in EtOH) with 30 µL of pH 6.8 phosphate buffer (0.067 M, pH 6.8), 40 µL of the enzyme tyrosinase (100 U/mL) and 40 µL of the substrate L-DOPA (2.5 mM) were added to each well. The reaction was incubated for 15 min at 24 °C and the absorbance was read at 492 nm. The results were expressed as IC_50_ (µg/mL), the final concentration of the plate ranged from 31.25 to 250 μg/mL, and the experiments were performed in triplicate. The values are reported as mean ± SD. Kojic acid was used as positive control.

### 2.10. Docking Studies

Docking simulations were carried for selected major compounds shown in Figure S1 (Supplementary Materials) obtained from *Gaultheria pumila* berries extract. First, the geometries and partial charges of every compound were fully optimized using the DFT method with the standard basis set PBE0/6-311 + g* [29,30] in Gaussian 09W software, version 9.0. Then, energetic minimizations and protonations or deprotonation (if applicable) were carried out using the LigPrep tool in Maestro Schrödinger suite v.11.8 (Schrödinger, LLC). Crystallographic enzyme structures of *Torpedo Californica* acetylcholinesterase (*Tc*AChE; PDBID: 1DX6 code) [31], human butyrylcholinesterase (*h*BuChE; PDBID: 4BDS code) [32], and the *Agaricus bisporus* mushroom tyrosinase (tyrosinase; PDBID: 2Y9X code) [33] were downloaded from the Protein Data Bank RCSB PDB [34] (for full description, see Supplementary Material).

### 2.11. Antiproliferative Activity

For the antiproliferative activity, 100 µL of cells with a density of 2500 (A549, HBL-100, HeLa, and SW1573) and 5000 (T-47D and WiDr) were added to each well. The plant extract was dissolved in DMSO at 100 mg/mL (400 times the desired maximum test concentration) and tested in triplicates at dilution ranges from 2.5 µg/mL to 250 µg/mL. Furthermore, control cells were exposed in DMSO (0.25% *v*/*v*, negative control). After 24 h, the extracts were incubated for 48 h and the cells were precipitated with 25 μL of ice-cold TCA (50% *w*/*v*), fixed for 60 min at 4 °C [35]. Finally, in each well, the optical density (OD) was measured at 530 nm and this was conducted with the Sulforhodamine B (SRB) assay and using the BioTeK Power Wave XS absorbance microplate reader. Values were corrected with the background OD of the wells containing the control. The antiproliferative activity of the extracts was expressed as the 50% reduction in cancer cell growth (GI_50_) and total growth inhibition (TGI).

### 2.12. Statistical Analysis

All the experiments were repeated at least three times. The results were expressed as mean ± standard deviation (SD) using GraphPad Prism 8. The comparison of results was performed using one-way analysis of variance (ANOVA), followed by Tukey’s HSD (Honest Significant Difference) test (*p* < 0.01).

## 3. Results and Discussion

### 3.1. UHPLC–MS Analysis of Gaultheria Pumila Extract

**Figure 2:**
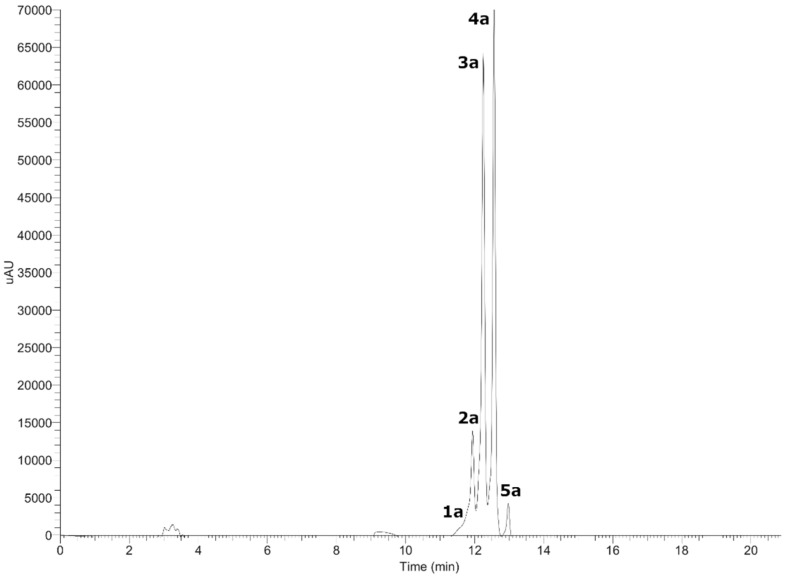
UHPLC-DAD chromatogram of anthocyanins from *Gaultheria pumila* at 520 nm.

**Figure 3:**
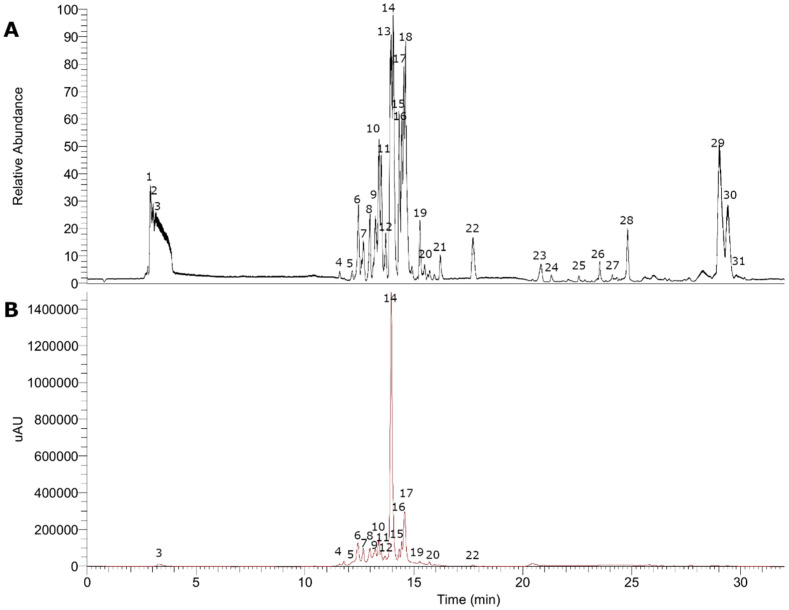
UHPLC chromatograms in the negative mode of *Gaultheria pumila*. (**A**) TIC chromatogram. (**B**) UV at 280 nm.

The fingerprinting of the enriched extract of *G. pumila* berries was investigated by means of UHPLC-high-resolution MS and DAD analysis. The negative mode was used for the identification of phenolic compounds, while the positive mode was used for anthocyanins. Some of the metabolites identified are reported for the first time in this species. In total, 36 metabolites were detected and tentatively identified including anthocyanins, phenolic acids, flavonoids, iridoids, terpenes, and fatty acids (see Figure 2 and Figure 3, Table 1, and Figure S1, Supplementary Material). A detailed analysis is depicted below.

#### 3.1.1. Anthocyanins

Five anthocyanins with λ max at 520 nm were detected in *G. pumila* berries using UHPLC-DAD-MS analysis in the positive mode (Figure 2). Peak 1a and 2a showed a common MS^2^ fragment, in agreement with delphinidin (C_15_H_11_O_7_^+^) at *m*/*z* = 303.30396 and *m*/*z* = 303.05667. Peak 1a was identified as delphinidin 3-*O-*arabinoside (C_20_H_19_O_11_^+^) and peak 2a as delphinidin 3-*O-*galactoside (C_21_H_21_O_12_^+^) [21]. The mass spectra peak 3a and 4a showed a diagnostic cyanidin MS^2^ ion at *m*/*z =* 287.06052 and *m*/*z =* 280.06049, and were identified as cyanidin-3-*O*-galactoside (C_21_H_21_O_11_^+^) and cyanidin-3-*O*-arabinoside (C_20_H_19_O_10_^+^) [21]. Peak 5a (*m*/*z =* 493.10559) with a daughter ion at *m*/*z* = 301.16412, in agreement with peonidin, was identified as peonidin-3-*O*-galactoside (C_22_H_23_O_11_^+^) [21]. From Chilean *Gaultheria* compounds, 1a, 2a, 3a, and 4a were previously identified on *G. phillyreifolia* [11], *G. poeppigii* [11], and *G. antartica* [14], and compounds 3a and 4a in *G. tenuifolia* berries [13]. To the best of our knowledge, compound 5a is reported for the first time in *G. pumila*. In addition, from Chilean *G. mucronata*, some undetected anthocyanins such as cyanidin-3-lathyroside and cyanidin-dipentoside were also identified [14].

#### 3.1.2. Flavonoids and Derivatives

Peak 3 with a [M-H]^−^ ion at *m*/*z* = 335.05420 was identified as the apoptotic furanoflavonoid pongapin (C_19_H_11_O_6_^−^) [36]. Peak 8 was identified as isoschaftoside [37]. Peak 11 and 14 with flavonol absorbance (λ_max_ at 254, 354 nm) and with anions at 479.08319 and 463.08838 were identified as myricetin-*O*-hexoside and myricetin-*O*-rhamnoside, previously identified from *G. phillyreifolia* [11], *G. poeppigii* [11] and *G. antartica* [14] berries. Peak 15, 16, and 17 were identified as quercetin derivatives (λ_max_ at 351 nm): quercetin 3-*O*-ribose (*m*/*z* = 433.07782), quercetin 3-*O*-arabinoside (*m*/*z* = 433.07776), and quercetin 3-O-xylose (*m*/*z* = 433.07773). Peak 15, 16, 17 showed common fragments at *m*/*z* = 301 (C_15_H_9_O_7_^−^) formed by the loss of pentose moiety. In addition, fragments at *m/z* = 151 (C_7_H_3_O_4_^−^) and *m*/*z* = 107 (C_6_H_3_O_2_^−^) formed by the retro Diels–Alder reaction of quercetin moiety were also identified. Peak 18 with a *m*/*z* = 447.09341 was identified as quercitrin (C_21_H_19_O_11_^−^) and showed a diagnostic fragment at *m*/*z* = 300.0275 due the loss of hexoside moiety, while peak 22 with a [M-H]^−^ ion at *m*/*z* = 301.03561 was identified as quercetin aglycone [38]. Quercetin derivatives have been previously reported from *G. phillyreifolia* [11], *G. poeppigii* [11], *G. mucronata* [14], and *G. antartica* [14]. Peak 19 with a [M-H]^−^ ion at *m*/*z* = 461.07294 was identified as luteolin 7-glucuronide according to characterized fragmentation. From Chinese *G. trichocada*, one luteolin derivative (luteolin-7-*O*-*β*-D-glucoside) was isolated and characterized [39]. Peak 23 was identified as procyanidin B-type dimer (C_27_H_45_O_13_^−^), previously detected on Chilean *Gaultheria* [11]. Peak 30 with a *m*/*z* = 343.08267 was identified as eupatorin (C_18_H_15_O_7_^−^), previously identified from the fruits of *Vitex agnus-castus* [40].

#### 3.1.3. Phenolic Acids

Peak 1 with a *m*/*z* = 377.08575 was identified as vaccihein A, previously isolated from the blueberry *Vaccinium ashei* [41]. Peak 4 showed a [M-H]^−^ ion at *m*/*z* = 377.08575 with a daughter ion at *m*/*z =* 191.05566 (quinic acid portion) and *m*/*z* = 135.04482 (formed by the decarboxylation of the caffeic acid portion) in agreements with a caffeoylquinic acid derivative (C_16 17_O_9_^−^) [42], also described on *G. phillyreifolia*, *G. poeppigii* [11], and *G. mucronata* [15]. Peak 5 was identified as the 1-*O*-protocatechuyl-beta-xylose: uralenneoside (C_12_H_13_O_8_^−^) [43]. Peak 6 (λ max 325 nm) showed a deprotonated molecule at *m*/*z* = 353.08804, an adduct ion at *m*/*z* = 707.18433 ([2M^−^H]^−^), and daughter ion at *m*/*z* = 191.05592 (quinic acid), and thus was tentatively identified as chlorogenic acid (C_16_H_17_O_9_^−^), previously isolated from *G. trichocada* [39]. Peak 7 showed a [M-H]^−^ ion at *m*/*z* = 417.08307 with a daughter ion at *m*/*z =* 163.03966 (loss of glucose) and *m*/*z =* 119.04950 (decarboxylated coumaric acid), and was identified tentatively as coumaric acid glucoside [44,45]. Peak 9 with a *m*/*z* = 335.07755 was identified as 5-*O*-Caffeoylshikimic acid, also reported in *G. phillyreifolia* and *G. poeppigii* [11]. Peak 10 with a deprotonated molecule at *m*/*z* = 551.14050 and with MS^2^ fragment at *m*/*z* = 179.03439 (caffeic acid) was assigned as a caffeic acid derivative (C_25_H_27_O_14_^−^) [46]. Peak 12 with a pseudo-molecular ion at *m*/*z* = 449.07294 (C_20_H_17_O_12_^−^) was identified as a methyl gallate derivative. From *Rhododendron ambiguum* (Ericaceae) fruits, methyl gallate glucosides were identified [47]. Peak 20 was identified as a galloyl derivative (*m*/*z* = 461.07297) [9]. In the MS^2^ spectrum, the precursor ion showed a fragment at *m*/*z* = 309.17999, formed by the loss of galloyl moiety at *m*/*z* = 151.00304 (C_7_H_3_O_4_^−^). Peak 26 with a deprotonated molecule at *m*/*z* = 487.08856 was identified as caffeoyl feruloyl tartaric acid (C_23_H_19_O_12_^−^) [48].

#### 3.1.4. Fatty Acids

Peak 24 with a *m*/*z* = 329.23383 was identified as pinellic acid (C_18_H_33_O_5_^−^) [49], peak 27 as octadecanedioic acid [50], and peak 28 with a deprotonated molecular ion at *m*/*z* 327.29071 as 2-hydroxyeicosanoic acid (C_20_H_39_O_3_^−^) [51]. Some fatty acids (stearic acid and palmitic acid) were identified and isolated previously from *Gaultheria itoana* [52].

#### 3.1.5. Terpenoids

Iridoids are monoterpenoids previously described in *Gaultheria* species. Coumaroyl iridoids were predominantly derived from *G. poeppigii* berries and *G. phillyreifolia* [11,12], and the secoiridoid Swertiamari and methoxygeniposidic acid, among others, were derived from *G. tenuifolia* [13]. Three iridoids were tentatively identified in *G. pumila*. Peak 2 with a deprotonated molecule at *m*/*z* 407.11966 (C_16_H_23_O_12_^−^) was identified as the iridoid glucoside unedide (6,7-dihydro-6β-hydroxymonotropein), also found in the Ericaceae family [53]. Peak 13 with a *m*/*z* = 535.14545 showed a characteristic fragment at *m*/*z* = 163.03975 (coumaroyl unit) and was identified as the iridoid *p*-coumaroyl monotropein isomer (C_25_H_27_O_13_^−^), previously reported in *G. phillyreifolia* and *G. poeppigii* [11]. Peak 21 was identified as the secoiridoid nuzhenal B (C_23_H_29_O_11_^−^), reported in the fruits of *Ligustrum lucidum* [54]. In addition, peak 25 with a *m*/*z* = 365.19760 was identified as the diterpenoid enmenol [55], peak 29 as the neo-clerodane diterpenoid scupolin I (C_24_H_35_O_8_^−^) [56], and peak 31 as the sesquiterpenoid dictamnoside M [57].

### 3.2. Total Phenolic, Flavonoid, and Anthocyanin Content, and Antioxidant Activity

*Gaultheria pumila* extract was assessed in vitro for total phenolic (TP), flavonoid (TF), and anthocyanin (TA) content, and antioxidant activity. The results are summarized in Table 2. To determine the antioxidant activity of a sample, it is important to combine several antioxidant experiments (e.g., DPPH, FRAP, and ORAC, among others) as these provide useful information on the interaction between radicals and samples, and also provide complementary information on reactive oxygen species (ROS) [23,58]. The results of TP, TF, TA, and the antioxidant activity were compared with previously published data related to *G. phillyreifolia*, *G. poeppigii* [11], *G. shallon* [59], and *G. erecta* [60] berries. From *G. pumila* berries, 36 metabolites were identified, some of which are flavonoids and derivatives, phenolic acids, iridoids, terpenes, and fatty acids. The results observed for TP and TF of *G. pumila* were 189.2 ± 0.2 mg gallic acid equivalent per g of extract and 51.8 ± 0.1 mg quercetin equivalent per g of extract, respectively. From purple fruits of *G. phillyreifolia and G. poeppigii*, the highest values for TP were 290.3 mg gallic acid equivalent per g and 184.4 mg quercetin equivalent per g for TF [11]. The Colombian *G. erecta* TP value was 881.3 mg gallic acid equivalent per g [60]. TA value for *G. pumila* was 47.3 ± 0.2 mg cyanidin-3-glucoside per g of extract. From Chilean *G. phillyreifolia* and *G. poeppigii*, reported values ranged from 8 to 87 mg cyanidin-3-glucoside equivalent per g [11]. In addition, previous studies from *G. pumila* berries collected from different locations showed similar results with our samples (59.42 mg cyanidin-3-glucoside per g) [16]. *G. pumila* berries showed better antioxidant activity in the ORAC (4251.6 ± 16.9 μM Trolox equivalents per 100 g of the dry plant) and DPPH (92.8 ± 0.1 μg/mL) assays. In contrast, the FRAP assay was lower (134.1 ± 0.1 μmol Trolox equivalent per g of dry plant) than those of Chilean *G. phillyreifolia* berries (6019.6 ± 42.0 μmol Trolox equivalent/g) [11]. Recently, TEAC, DPPH, and CUPRAC experiments were investigated from *G. poeppigii* samples [12]. In the ORAC assay, the *G. pumila* value was 4251.6 μmol Trolox equivalent per g of dry plant. In addition, *G. poeppigii* berries showed the best results in the ORAC (2412–6145 μmol Trolox equivalent per g of dry plant) and DPPH (IC_50_ from 10.3 ± 0.5 μg/mL) experiments [11]. The antioxidant activity was also evaluated for Chilean *G. mucronata* (28.9 ± 4.2 μmol TEAC/g) and *G. antarctica* (20.3 ± 5.2 μmol TEAC/g) using Trolox equivalent antioxidant capacity (TEAC) assays [14].

Previous studies with others berries such as calafate (*Berberis microphylla*) and zarzaparilla (*Ribes magellanicum*) showed higher antioxidant capacities estimated by TEAC; this is attributed to the presence of higher concentrations of anthocyanins in the fruits [14]. In similar studies, the blueberry *Vaccinium myrtillus* was evaluated regarding its antioxidant capacity and the total phenolic content. The antioxidant activity was higher in the colored berries than in the white berries and the total phenolic content was also higher [61]. The Maqui berry (*Aristotelia chilensis*) has demonstrated a great proportion of polyphenolic compounds (phenolic acids, flavonoids, and anthocyanins) as antioxidant agents [62]. Finally, it can be considered that the antioxidant capacity observed in the *G. pumila* study may be useful as a protection against free radicals; these results could be explained by the presence of different types of phenolic compounds with antioxidant properties and by the comparison based on other berries.

### 3.3. Enzymatic Inhibitory Activity

*Gaultheria pumila* berries were assessed in vitro for cholinesterase and tyrosinase inhibitory potential. To the best of our knowledge, no previous reports regarding anti-enzymatic potential have been conducted in this species. The results are summarized in Table 3 and are expressed as IC_50_ values (µg/mL). The use of berries has been important over the years to prevent neurodegenerative diseases due to their high content of phenolic compounds [25]. *G. pumila* showed moderate activity against AChE (IC_50_ = 7.7 ± 0.3 µg/mL), BChE (IC_50_ = 34.5 ± 0.5 µg/mL), and tyrosinase (IC_50_ = 3.3 ± 0.2 µg/mL) in the enzymatic assays. Regarding the metabolites identified in the extract of *G. pumila*, some reports indicated that, for example, cyanidin-3-*O*-glucoside showed properties against mono amine oxidase-A, tyrosinase, and fatty acid amide hydrolase (FAAH) enzymes, whereas no activity was found against AChE [63]. In docking studies, chlorogenic acid was demonstrated to inhibit cholinesterase enzymes [64]. Isoquercitrin exhibited potent alpha-glucosidase, tyrosinase, and xanthine oxidase enzyme-inhibitory activities [65]. Delphinidin-3-*O*-glucoside, cyanindin-3-*O*-glucoside, peonidin-3-*O*-glucoside, and malvidin-3-*O*-glucoside were the principal anthocyanin detected in red wines from Serbia vineyards, which were related to AChE inhibitory activity [66]. Quercetin derivatives (quercetin-3-*O*-glucoside) and other flavonoids were identified as kaempferol and isorhamnetin derivates from *Hippophae rhamnoides* berries, which have demonstrated AChE and BChE inhibitory activities [67]. Regarding chlorogenic acid, BChE inhibitory activity has been reported [68]. From the Gaultheria species, tyrosinase inhibitory activity has been reported in *Gaultheria erecta,* suggesting properties against skin aging [60]. Conversely, BChE inhibitory activity (35.52 ± 1.17 μg/mL) has been reported for *Gaultheria trichophylla* [69]. These reports highlight the importance of metabolites contained in *Gaultheria* fruits that could be suitable for use in the prevention of neurodegenerative diseases.

### 3.4. Docking Studies

Five selected major compounds according to the UHPLC chromatogram (Figure 3) obtained from *G. pumila* berries extract as well as the known cholinesterase and tyrosinase inhibitors, galantamine and kojic acid, respectively, were subjected to docking assays in the acetylcholinesterase catalytic site, butyrylcholinesterase catalytic site, and tyrosinase catalytic site in order to rationalize their pharmacological results and analyze their protein molecular interactions in light of the experimental inhibition activities obtained (Table 3). The best docking binding energies expressed in kcal/mol of each selected compound are shown below.

#### 3.4.1. Acetylcholinesterase (TcAChE) Docking Results

Table 4 shows binding energies of Scupolin I, Unedide, Nuzhenal B, Myricetin *O*-rhamnoside, and Luteolin 7-glucuronide. All the aforementioned compounds displayed good energy descriptors over the acetylcholinesterase enzyme. Some of them showed binding energies in a similar range compared to galantamine and in case of Nuzhenal B, Myricetin 3-rhamnoside, and Luteolin 7-glucuronide, a better energy profile was even obtained. The latter would explain the higher potency (less IC_50_ value) over acetylcholinesterase than as presented by the berries extract over butyrylcholinesterase (IC_50_ = 34.52 ± 0.05). Even though galantamine exhibited a binding energy of −12.989 kcal/mol and Nuzhenal B or Myricetin *O*-rhamnoside possessed slightly better energy parameters, the fact that the aqueous berries extract contained other active compounds lead into a competition among all of them for the acetylcholinesterase catalytic site; a refined result cannot be obtained. Nonetheless, it is clear that the good energies presented by these major compounds in the extract could be the responsible for the strong inhibitory effect shown over the enzyme. In terms of molecular interactions among each compound and the residues of the catalytic site, mainly all derivatives performed hydrogen bond interactions with the catalytic amino acids.

**Figure 4:**
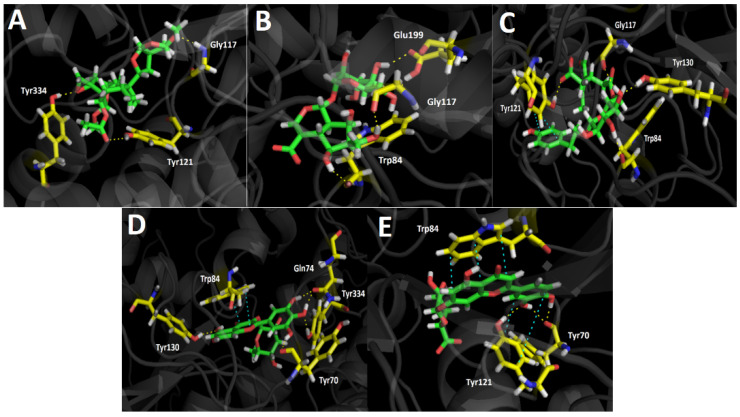
Predicted binding mode and predicted intermolecular interactions of the selected major compounds in *Gaultheria pumila* berries extract and the residues of the *Torpedo Californica* acetylcholinesterase (*Tc*AChE) catalytic site. Yellow dotted lines indicate hydrogen bond interactions and cyan dotted lines represent π–π interactions. (**A**) Scupolin I in the catalytic site; (**B**) Unedide in the catalytic site; (**C**) Nuzhenal B in the catalytic site; (**D**) Myricetin *O*-rhamnoside in the catalytic site; and (**E**) Luteolin 7-glucuronide in the catalytic site.

In this sense, the compounds that showed better energies also exhibited a greater amount of hydrogen bond interactions. Scupolin I performed three different hydrogen bond interactions (Figure 4A), all of them are carried out by oxygen atoms; one through a methoxy group and the other two through the epoxide function, and the ester carbonyl resembled its structure. Unedide also showed three hydrogen bond interactions where its hydroxyl groups are implied; this allows a protein–inhibitor complex stabilization through the amino acids of Trp84, Gly117, and Glu199 (Figure 4B). The good binding energy values shown by Nuzhenal B and Myricetin *O*-rhamnoside may be supported by the higher amount of hydrogen bond interactions performed, as well as the extra π–π interaction that both derivatives carry out into the acetylcholinesterase catalytic site. Nuzhenal B exhibited three hydrogen bond interactions due to the hydroxyl groups at the glycoside moiety and also another specific hydrogen bond interaction between the deprotonated carboxylic group (carboxylate) of its branched aliphatic chain and the amino acid Tyr121 (Figure 4C). Moreover, the π–π interaction mentioned above is also performed with Tyr121 through the phenolic ring of this residue and the presence of the phenolic ring into the Nuzhenal B structure. In the case of Myricetin *O*-rhamnoside, the largest number of hydrogen bond interactions are made by the hydroxy functions at the pyrogallol framework with the residues of Tyr70, Gln74, and Tyr334 of the acetylcholinesterase. In addition, another hydrogen bonding can be noted between a hydroxyl group of the 4H-chromen-4-one core and the amino acid Tyr130. The π–π interaction is carried out between the 4H-chromen-4-one and the aromatic indole ring of Trp84 (Figure 4D). Luteolin 7-glucuronide exhibited a good energy value of −13.757 kcal/mol (Table 4). It is noteworthy that even though this derivative showed only three hydrogen bonding interactions, the fact that it also performed two different π–π interactions, one through its 4H-chromen-4-one moiety with the Trp84 amino acid and the other by the catechol ring and the Tyr121 residue, most likely contributes to its energy profile (Figure 4E).

#### 3.4.2. Butyrylcholinesterase (hBuChE) Docking Results

**Figure 5:**
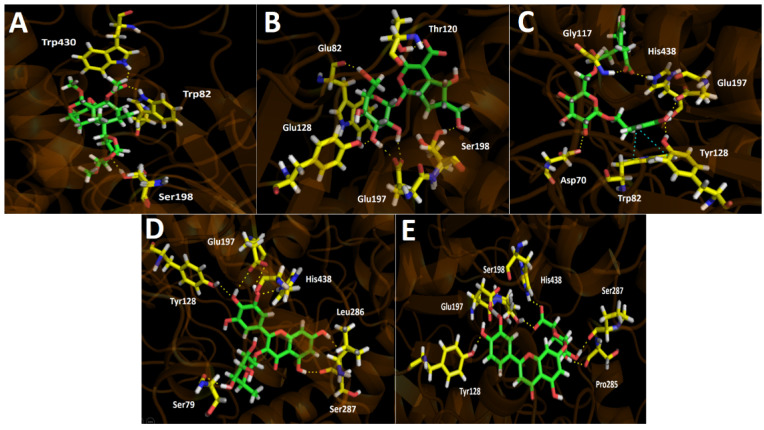
Predicted binding mode and predicted intermolecular interactions of the selected major compounds in *Gaultheria pumila* berries extract and the residues of the human butyrylcholinesterase (*h*BuChE) catalytic site. Yellow dotted lines indicate hydrogen bond interactions and cyan dotted lines represent π–π interactions. (**A**) Scupolin I in the catalytic site; (**B**) Unedide in the catalytic site; (**C**) Nuzhenal B in the catalytic site; (**D**) Myricetin *O*-rhamnoside in the catalytic site; and (**E**) Luteolin 7-glucuronide in the catalytic site.

Binding energies from docking assays over butyrylcholinesterase (*h*BuChE) of the selected major compounds from *Gaultheria pumila* berries extract showed to be poorer compared to those in acetylcholinesterase (*Tc*AChE). Almost all compounds present a good binding energy profile, except for Scupolin I as in acetylcholinesterase (Table 4). Although tested derivatives show slightly better energies compared to galantamine, not a wide difference is contemplated, suggesting the reason for the closer IC_50_ values between galantamine and the *G. pumila* berries extract in our inhibition assays. In fact, both half-maximal inhibitory concentrations are in the same order of magnitude (IC_50_ = 34.52 ± 0.05 for the extract and IC_50_ = 3.82 ± 0.08 for galantamine). As in acetylcholinesterase docking assays, the intermolecular interactions in the butyrylcholinesterase catalytic site that predominates are hydrogen bond interactions and a π–π interaction in the case of Nuzhenal B. Scupolin I binding descriptors over butyrylcholinesterase share some similarities to those showed in acetylcholinesterase docking results for this derivative. Indeed, Scupolin I shows three different hydrogen bond interactions performed by the oxygen atoms of one of the alicyclic tetrahydrofuran rings contained in its structure and the esther carbonyl with Ser198, Trp82, and Trp430 (Figure 5A). Unedide, which exhibited a better binding energy than Scupolin I, also presented more hydrogen bond interactions, mainly through the hydrogen atoms of the different hydroxyl functions at its glycoside moiety with Trp82 and Tyr128, as well as with the oxygen atoms of the deprotonated carboxylate of Glu197. Furthermore, it can be seen that two other hydrogen bond interaction were performed by a hydroxyl group and a carboxylate (contained at the hexahydrocyclopenta[*b*]pyrane core of Unedide) with Ser198 and Thr120, respectively (Figure 5B).

Due to the fact that Nuzhenal B bore a glycoside moiety in its structure, this derivative possesses the ability to carry out hydrogen bond interactions through the hydroxyl groups present at this core, but only one of these interactions are displayed by a hydroxy group of this framework with the amino acid Asp70 (Figure 5C). Given the above, Nuzhenal B arranged into the catalytic site through hydrogen bondings performed by a phenolic group and a carboxylate function of its structure. The residues implied in the four interactions mentioned above are Tyr128, Glu187, His438, and Gly117 (Figure 5C). Moreover, Nuzhenal B showed a π–π interaction with the indole ring of Trp82 and the phenolic moiety of its structure as already aforementioned. Myricetin *O*-rhamnoside and Luteolin 7-glucuronide, which share the 4*H*-chromen-4-one framework in their structures, also showed the best binding energy profiles. The latter could be attributed due to the fact that both derivatives showed more than five hydrogen bond interactions with the different residues of the butyrylcholinesterase catalytic site, either through the hydroxy functions at the pyrogallol framework in Myricetin *O*-rhamnoside or through the hydroxy groups at the catechol ring in Luteolin 7-glucuronide (Figure 5D,E). Additionally, the presence of glycosidic portions in both derivatives also allowed it to perform hydrogen bond interactions, but this core in Luteolin 7-glucuronide becomes more relevant as it carried out four interactions through its hydroxyl functions, alongside a special hydrogen bonding with the residue Ser198 and the deprotonated carboxylate that this moiety possesses (Figure 5E).

#### 3.4.3. Tyrosinase Docking Results

**Figure 6:**
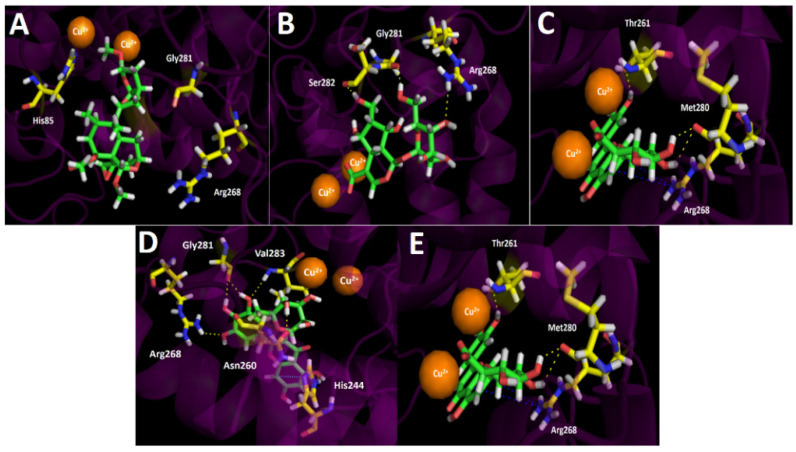
Predicted binding mode and predicted intermolecular interactions of the selected major compounds in *Gaultheria pumila* berries extract and the residues of the *Agaricus bisporus* mushroom tyrosinase catalytic site. Yellow dotted lines indicate hydrogen bond interactions, cyan dotted lines represent π–π interactions, red dotted lines indicate salt bridge interactions, and blue dotted lines indicate π-cation interactions. (**A**) Scupolin I in the catalytic site; (**B**) Unedide in the catalytic site; (**C**) Nuzhenal B in the catalytic site; (**D**) Myricetin *O*-rhamnoside in the catalytic site; and (**E**) Luteolin 7-glucuronide in the catalytic site.

Inhibition assays of *Gaultheria pumila* berries extract over tyrosinase turned out to be more potent than the known inhibitor kojic acid by three-folds. Even though the precise reaction mechanism of tyrosinase is unclear and no information is available about the binding mode of many substrates, the higher potency shown by our berries extract could be explained by the docking results summarized in Table 4 and Figure 6.

Regarding the intermolecular interactions of the selected major compounds obtained from the UHPLC chromatogram, docking descriptors suggest that the main inhibitory activity would lie in Nuzhenal B, Myricetin *O*-rhamnoside, and Luteolin 7-glucuronide derivatives. Indeed, Scupolin I docking assays over tyrosinase, just like in acetylcholinesterase and butyrylcholinesterase, showed a deficient binding energy of −4.303 kcal/mol and no contributing interactions were present among the residues of the catalytic site and Scupolin I structure, except with some hydrophobic amino acids. This phenomenon could be due to the lack of chemical groups by Scupolin I capable of performing other sorts of interactions such as π–π, T-shaped, or π-cation interaction. Thus, probably this compound would not contribute to the enzyme inhibition in a significant manner, even if it is in high proportion in the extract. As tyrosinase bore two copper cations leading to a binuclear copper-binding site in which three histidine residues coordinate each ion (His61, His85, and His94 for one copper cation, and His259, His263, and His296 for the other), these metal atoms could play a key role in the stabilization of some protein–inhibitor complexes with the different substrates. In this sense, Unedide not only carried out three hydrogend bond interactions among its hydroxyl groups and the residues of Gly281, Ser282, and Arg268, but also a salt bridge between one of the copper ions and the deprotonated carboxylate at its hexahydrocyclopenta[*b*]pyrane core (Figure 6B). In the same way, Nuzhenal B and Luteolin 7-glucuronide also exhibited salt bridge interactions between their carboxylate groups and one copper ion. The salt bridges aforementioned by Nuzhenal B and Luteolin 7-glucuronide are possible due to the fact that the first one bore a carboxylate function in its branched aliphatic chain and the second one on its glycoside moiety. Conversely, Myricetin *O*-rhamnoside, due to a lack of a carboxylic acid group on its structure, would be unable to perform a salt bridge interaction with the tyrosine catalytic site (Figure 6D). In addition to the salt bridge interactions of the three derivatives already described, it should be noted that docking assays revealed that Nuzhenal B also carried out four hydrogen bond interactions among the hydroxyl groups at the phenol moiety and at the glycoside core, as well as with the oxygen atom of the esther carbonyl. The organic functions of Nuzhenal B mentioned above interacted with His85, Glu322, His244, and Asn260. Myricetin *O*-rhamnoside showed hydrogen bond interactions with His244, Asn260, Arg268, Gly281, and Val283, and also a π–π interaction with the imidazole ring of His244 was performed. This last interaction could be improving the affinity of this compound for the tyrosinase catalytic site. Luteolin 7-glucuronide was the only derivative that showed, in addition to the hydrogen bondings, a π-cation interaction between its 4*H*-chromen-4-one framework and the cationic protonated side chain of Arg268 (Figure 6E).

In order to summarize the information, the main interactions of the compounds with the best binding energy profile were performed, displayed in a two-dimensional diagram below (Supplementary Material).

### 3.5. Antiproliferative Activity

The antiproliferative activity of *G. pumila* berries was evaluated against six human solid tumor cell lines. The results show that *G. pumila* was not able to induce antiproliferative effects under the standard protocol conditions (GI_50_ and TGI > 250 μg/mL in all cell lines). For some compounds contained in the extract, the literature reports activity against cancer cell lines. Several anthocyanins showed cardioprotective, neuroprotective, antidiabetic, and anticancer effects [70]. Anthocyanins such as cyanidin-3-*O*-glucoside and delphinidin-3-*O*-glucoside prevent tumor progression by inhibiting angiogenesis through VEGFR-2 downregulation using cell models [71]. Furthermore, it has been reported that these compounds, which are also present in blackcurrant berries, exhibited antiproliferative properties on several solid tumor cancer cells [72]. In other studies, chlorogenic acid was attributed to have effects against breast cancer cell growth [73], quercitrin to have antiproliferative and apoptotic effects on lung cancer cells by modulating the immune response [74], and aglycone quercetin to be able to exert antitumor action by inhibiting cell proliferation, inhibiting angiogenesis, and preventing the progression of metastasis against various cancers [75]. For example, previous studies have found that the combination of quercetin with ionizing radiation showed a greater response against colon cancer stem cells. In addition, the combination of quercetin with resveratrol in a dermal gel favored penetration into deeper layers of the skin and thus can serve as a promising component to be used in formulations for the treatment of skin cancer [76,77]. Other results showed that quercetin exhibits inhibitory activity against the growth of metastatic ovarian cancer cells [78]. In another study, scutellarin derivatives were demonstrated to be active against Jurkat, HCT-116, and MDA-MB-231 cancer cell lines [79]. Our results showed no cytotoxic activity against A549 (lung), HBL-100 (breast), HeLa (cervix), SW1573 (lung), T-47D (breast), and WiDr (colon) cell lines. However, these previous results showed that some individual compounds contained in *G. pumila* berries could be used for the prevention of cancer due to its cytotoxic activities.

## 4. Conclusions

The antioxidant, antiproliferative, and enzyme inhibition potential, and the chemical fingerprinting of *G. pumila* berries were investigated for the first time. High resolution mass spectrometry (UHPLC-DAD-Orbitrap-MS) was used to detect 36 metabolites. To the best of our knowledge, this is the first investigation on the inhibitory activities (against cholinesterase and tyrosinase) and antiproliferative screening of these berries, updating the knowledge on the biological profile of this interesting species. The results from the enzyme inhibition studies demonstrated a moderate inhibition and did not show antiproliferative activity. Docking studies of the selected major compounds indicated that hydrogen bond interactions and a π–π interaction are predominant over acetylcholinesterase and butyrylcholinesterase, while a salt bridge interaction is predominant in the case of tyrosinase. Bioassay-guided fractionation and isolation of major compounds are needed to further characterize the molecule(s) responsible for the selected biological activities.

## Figures and Tables

**Table 1:** Tentative identification of secondary metabolites from *Gaultheria pumila* fruits.

Peak	RT	λ Max (nm)	Elemental Composition [M + H]^+^ [M − H]^−^	Measured Mass (*m*/*z)*	Theorical Mass (*m-z*)	Accuracy (ppm)	MS^n^ Ions	**Tentative Identification**
1a	11.45	234–282–520	C_20_H_19_O_11_^+^	435.10989	435.09219	40.685	303.05676, 247.12254, 178.13483, 155.15498, 130.00858	Delphinidin-3-*O*-arabinoside
2a	11.94	239–522	C_21_H_21_O_12_^+^	465.12381	465.10275	45.144	303.05667, 247.12254, 183.10243, 130.15967	Delphinidin 3-*O*-galactoside *
3a	12.26	280–521	C_21_H_21_O_11_^+^	449.12598	449.10784	40.394	287.06052, 247.12250, 171.09984, 147.04466	Cyanidin 3-*O*-galactoside *
4a	12.58	281–517	C_20_H_19_O_10_^+^	419.11234	419.09727	35.949	287.06049, 209.04585, 147.04463	Cyanidin-3-*O*-arabinoside *
5a	12.99	238–309–516	C_22_H_23_O_11_^+^	463.10559	463.12349	−38.648	301.16412, 225.81639, 171.09319	Peonidin-3-*O*-glucoside
1	2.94	232	C_18_H_17_O_9_^−^	377.08575	377.08781	−2.542	279.19812, 218.78490, 162.83897	Vaccihein A
2	3.06	232	C_16_H_23_O_12_^−^	407.11966	407.11950	3.089	295.51428, 156.00233, 119.60213	Unedide
3	3.38	256	C_19_H_11_O_6_^−^	335.05420	335.05611	−2.431	268.80099, 215.03267, 160.84152	Pongapin
4	11.58	234–297	C_16_ H_17_O_9_^−^	353.08820	353.08781	4.224	314.02936, 191.05566, 135.04482, 109.02880	Caffeoylquinic acid derivative
5	12.17	236–282	C_12_H_13_O_8_^−^	285.06189	285.06159	4.898	219.44096, 167.07101, 108.02096	Uralenneoside
6	12.47	236–299	C_16_H_17_O_9_^−^	353.08804	353.08781	3.771	707.18433, 191.05592	Chlorogenic acid
7	12.67	236–282–517	C_20_H_17_O_10_^−^	417.08307	417.08272	3.469	163.03966, 128.04340, 119.04950	Coumaric acid glucoside
8	12.98	236–312	C_26_H_27_O_14_^−^	563.14105	563.14063	2.696	281.06680, 191.05582, 115.91994	Isoschaftoside
9	13.25	237–323	C_16_H_15_O_8_^−^	335.07755	335.07724	4.196	213.96364, 179.03458, 135.04459	5-*O*-Caffeoylshikimic acid
10	13.39	238–326	C_25_H_27_O_14_^−^	551.14050	551.14063	1.757	179.03439, 135.04460	Caffeic acid derivative
11	13.50	254–354	C_21_H_19_O_13_^−^	479.08319	479.08311	2.448	317.09708, 289.08298, 213.96367, 115.91998	Myricetin-*O*-hexoside
12	13.70	238	C_20_H_17_O_12_^−^	449.07294	449.07255	3.313	319.04620, 183.02954, 134.89410	Methyl gallate derivative
13	13.96	241–304	C_25_H_27_O_13_^−^	535.14545	535.14571	1.556	357.11948, 163.03975, 119.04951	*p*-coumaroyl monotropein isomer
14	14.05	255–352	C_21_H_19_O_12_^−^	463.08838	463.08820	2.759	385.11417, 281.06665, 163.03955	Myricetin-*O*-rhamnoside
15	14.34	243–351	C_20_H_17_O_11_^−^	433.07782	433.07763	2.961	300.02753, 279.05096, 151.00316, 138.07101, 107.01299	Quercetin 3-*O*-ribose
16	14.41	255–351	C_20_H_17_O_11_^−^	433.07776	433.07763	2.822	300.02744, 151.03909 134.89404, 115.91995	Quercetin 3-*O*-arabinoside *
17	14.53	255–347	C_20_H_17_O_11_^−^	433.07773	433.07763	2.753	301.03571, 263.05963, 151.00317, 115.92000	Quercetin 3-*O*-xyloside
18	14.62	245–317	C_21_H_19_O_11_^−^	447.09341	447.09329	2.734	300.02750, 251.05937, 115.92000, 151.00310	Quercitrin
19	15.28	244–339	C_21_H_17_O_12_^−^	461.07294	461.07255	3.227	285.04007, 174.95566, 151.03946, 132.86728, 108.02094	Luteolin-7-glucuronide
20	15.49	244	C_21_H_17_O_12_^−^	461.07297	461.07255	3.292	309.17999, 195.96376 151.00304, 123.04469	Galloyl derivative
21	16.21	244	C_23_H_29_O_11_^−^	481.17169	481.17154	2.602	255.82225, 160.84169, 115.92001	Nuzhenal B
22	17.71	248	C_15_H_9_O_7_^−^	301.03555	301.03538	4.222	285.04120, 236.97911, 151.00301, 121.02882, 107.01293	Quercetin
23	20.86	245	C_27_H_45_O_13_^−^	577.28729	577.28657	3.157	450.53427, 425.21106, 207.85930	Procyanidin B-type dimer
24	21.32	247	C_18_H_33_O_5_^−^	329.23383	329.23335	4.797	197.80783, 160.84171, 122.02425	Pinellic acid
25	22.58	250	C_20_H_29_O_6_^−^	365.19760	365.19696	4.751	314.38947, 221.08304, 153.09119	Enmenol
26	23.56	254–290	C_23_H_19_O_12_^−^	487.08856	487.08820	3.054	427.03278, 311.22318, 191.10770	Caffeoyl feruloyl tartaric acid
27	24.13	255	C_18_H_33_O_4_^−^	313.23877	313.23843	4.578	284.97467, 174.95573, 149.92661, 116.95750	Octadecanedioic acid
28	24.81	257	C_20_H_39_O_3_^−^	327.29071	327.29047	4.089	266.28955, 206.63348, 136.34750	2-Hydroxyeicosanoic acid
29	29.03	258	C_24_H_35_O_8_^−^	451.23380	451.23374	2.561	297.15295, 265.14801, 235.09744	Scupolin I
30	29.41	258	C_18_H_15_O_7_^−^	343.08267	343.08233	4.199	279.16360, 265.14801, 233.08183, 162.83871	Eupatorin
31	29.81	258	C_27_H_47_O_14_^−^	595.28961	595.29713	−10.789	253.98213, 198.76825, 144.82085	Dictamnoside M

Abbreviations: RT = retention time and * = identified using authentic compounds.

**Table 2:** Total phenolic, flavonoid, and anthocyanin content, and antioxidant activity of *Gaultheria pumila* berries.

Assay	*Gaultheria pumila*	Standard
Total phenolics ^A^	189.2 ± 0.2	-
Total flavonoids ^B^	51.8 ± 0.1	-
Total anthocyanin ^C^	47.3 ± 0.2	-
FRAP ^D^	134.1 ± 0.1	-
ORAC ^E^	4251.6 ± 16.9	-
DPPH ^F^	92.8 ± 0.1	Gallic acid: 0.55 ± 0.1

All values are expressed as means ± SD (*n* = 3). ^A^ is expressed in mg gallic acid equivalent per g of extract. ^B^ is expressed in mg quercetin equivalent per g of extract. ^C^ is expressed in mg cianydin-3-glucoside equivalent per g of extract. ^D^ is expressed in μmol Trolox equivalent per g of dry plant. ^E^ is expressed in μmol Trolox equivalent per g of dry plant. ^F^ is expressed as IC_50_ in µg of extract or standard per mL. Abbreviations: FRAP, ferric reducing/antioxidant power; ORAC, oxygen radical absorbance capacity; ABTS, 2, 20-azino-bis (3-ethylbenzothiazoline-6-sulfonic acid; and DPPH, 2, 2-diphenyl-1-picryl-hydrazyl-hydrate.

**Table 3:** Enzymatic inhibitory activity of *Gaultheria pumila* berries.

Assay	AChE Inhibition IC_50_ (µg/mL)	BChE Inhibition IC_50_ (µg/mL)	Tyrosinase Inhibition IC_50_ (µg/mL)
***Gaultheria pumila***	7.7 ± 0.3	34.5 ± 0.5	3.3 ± 0.2
**Galanthamine**	0.3 ± 0.3	3.82 ± 0.2	-
**Kojic acid**	-	-	10.0 ± 1.9

All values are expressed as means ± SD (*n* = 3). Abbreviations: AChE, acetylcholinesterase and BChE, butyrylcholinesterase.

**Table 4:** Binding energies obtained from docking experiments of selected major compounds in *Gaultheria pumila* berries extract, as well as the known inhibitors galantamine and kojic acid over acetylcholinesterase (*Tc*AChE), butyrylcholinesterase, (*h*BChE) and tyrosinase accordingly.

Compound	Binding Energy (kcal/mol) Acetylcholinesterase	Binding Energy (kcal/mol) Butyrylcholinesterase	Binding Energy (kcal/mol) Tyrosinase
Scupolin I (29)	−10.582	−7.361	−4.303
Unedide (2)	−12.081	−10.972	−10.643
Nuzhenal B (21)	−15.289	−11.079	−10.576
Myricetin *O*-rhamnoside (14)	−15.175	−11.581	−10.528
Luteolin 7-glucuronide (19)	−13.757	−11.777	−11.323
Galantamine	−12.989	−7.125	-
Kojic acid	-	-	−6.050

## Data Availability

Data from the study is available on the request from a corresponding author.

## Supplementary Materials

The following are available online at https://www.mdpi.com/article/10.3390/metabo11080523/s1, Figure S1: High resolution UHPLC-PDA-Orbitrap-MS identification of metabolites in *Gaultheria pumila* fruits, Figure S2: Compounds subjected to docking assays into the corresponding catalytic sites of acetylcholinesterase, butyrylcholinesterase, and tyrosinase, Figure S3: Two-dimensional diagram.
