# Supplementary material for: UHPLC-MS Chemical Fingerprinting and Antioxidant, Antiproliferative, and Enzyme Inhibition Potential of Gaultheria pumila Berries
Source: Metabolites. 2021 Aug 7;11(8):523. doi: 10.3390/metabo11080523 (PMC8401902; doi:10.3390/metabo11080523)
Supplement: Supplementary file 1 [file metabolites-11-00523-s001.zip › metabolites-1326121-supplementary.pdf]

Figure S1. High resolution UHPLC-PDA-Orbitrap-MS identification of metabolites in *Gaultheria pumila* fruits.

MS experiment of Cyanidin-3-*O*-arabinoside *m/z* = 419.11234 (Peak 4a).

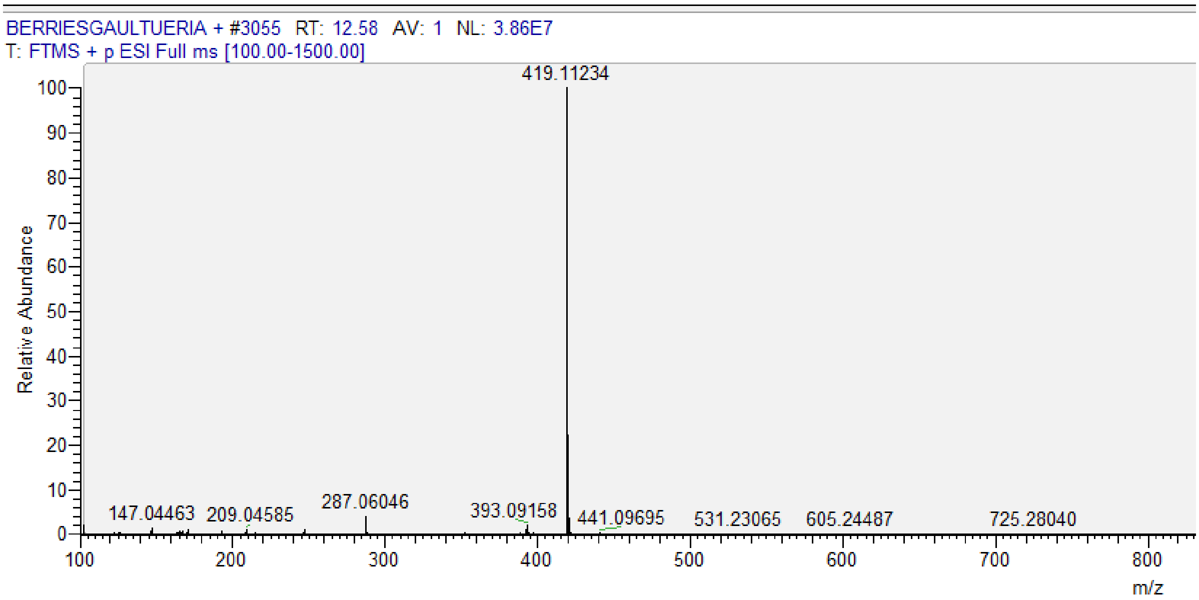

MS and MS-MS experiment of 4-*O*-Caffeoylquinic acid *m/z* = 353.08820 (Peak **4**)

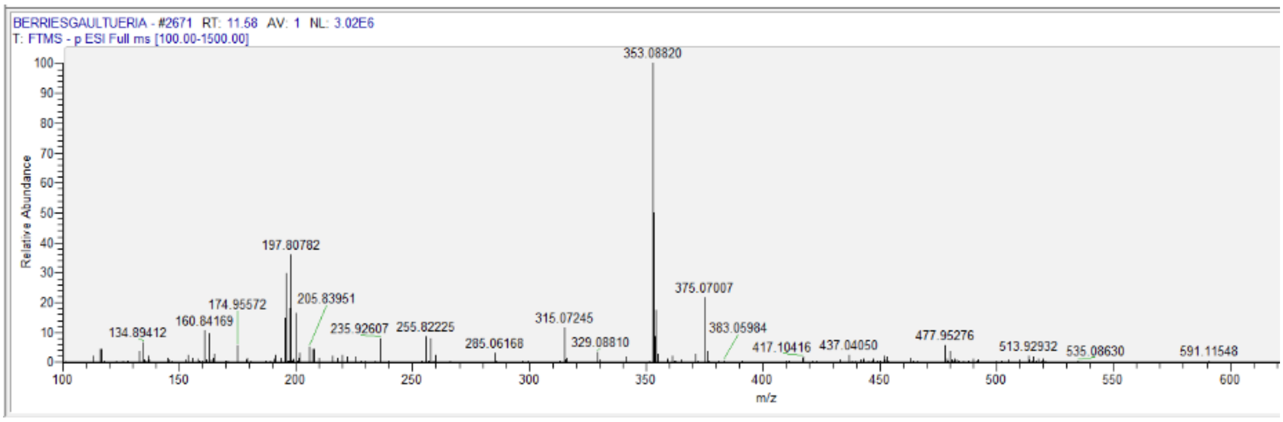

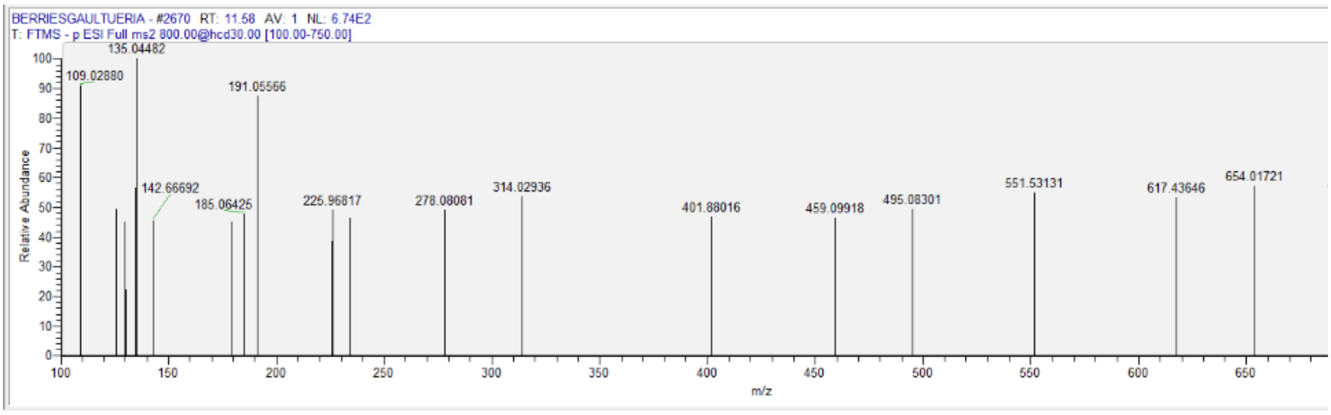

MS experiment of Chlorogenic acid *m/z* = 353.08801 (Peak 6).

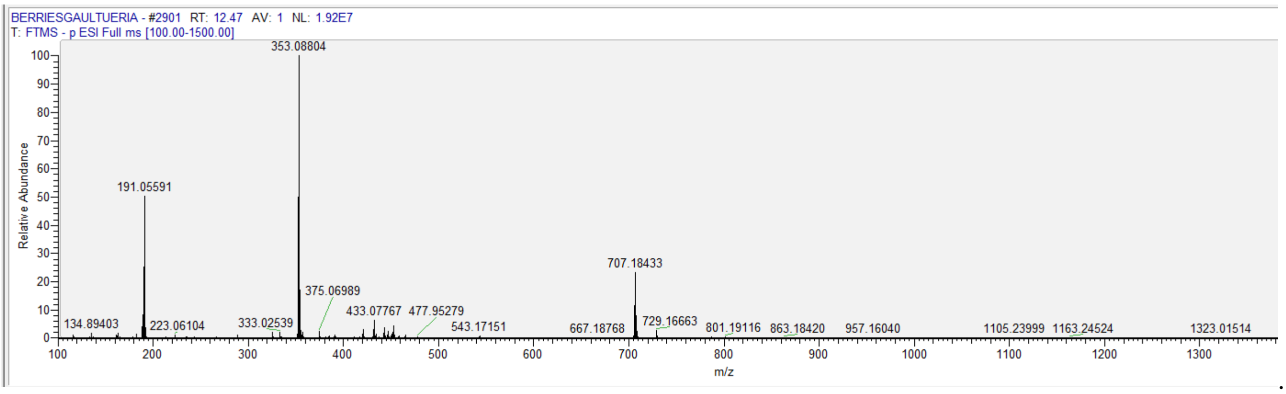

MS and MS-MS experiment of Coumaric acid glucoside *m/z* = 417.08307 (Peak 7).

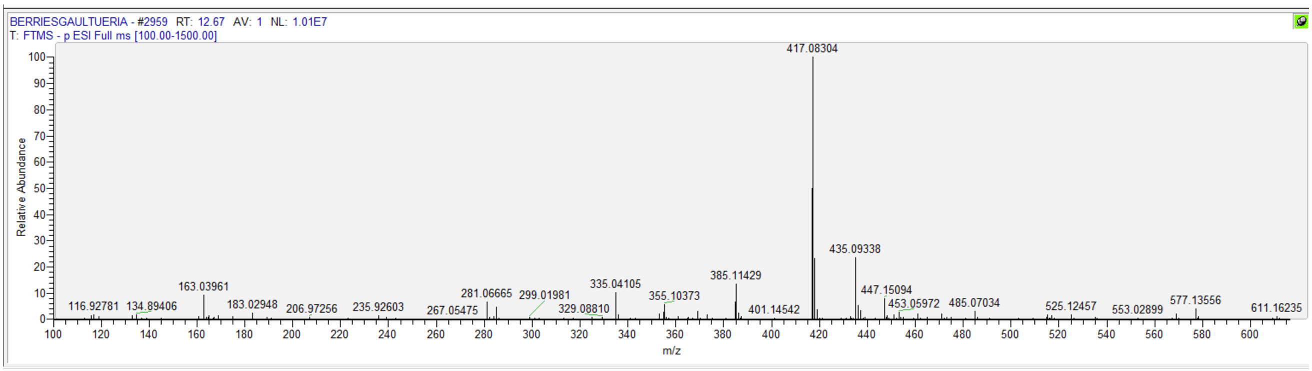

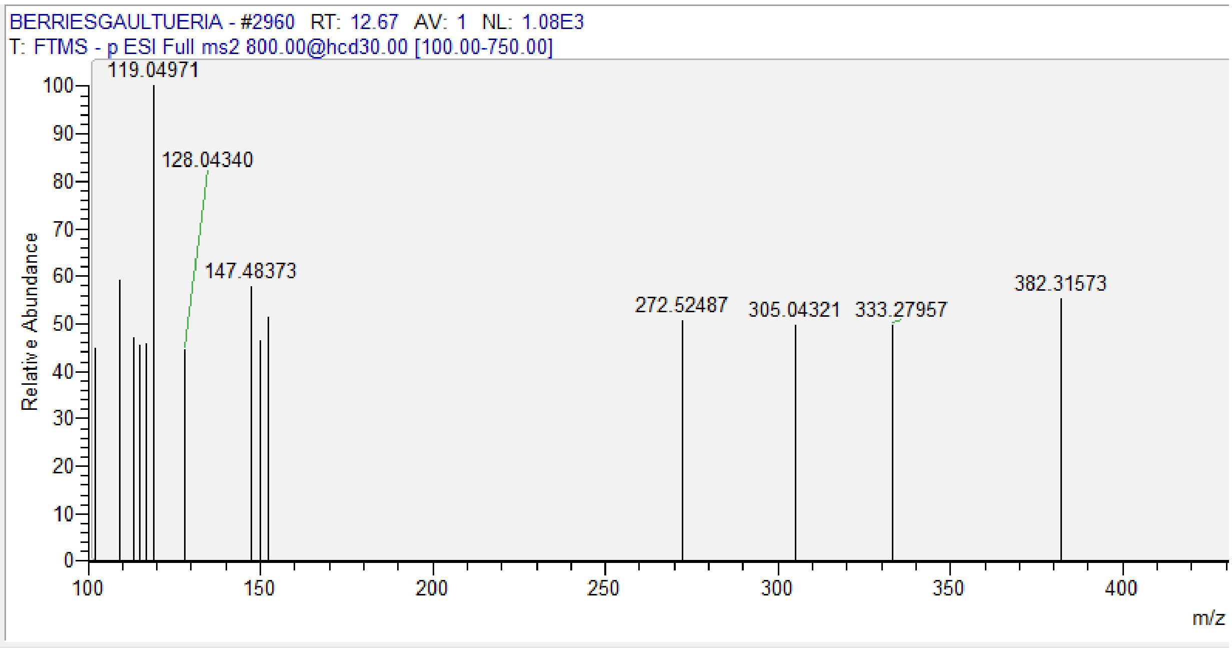

MS and MS-MS experiment of caffeic acid derivative m/z = 551.14050 (Peak 10).

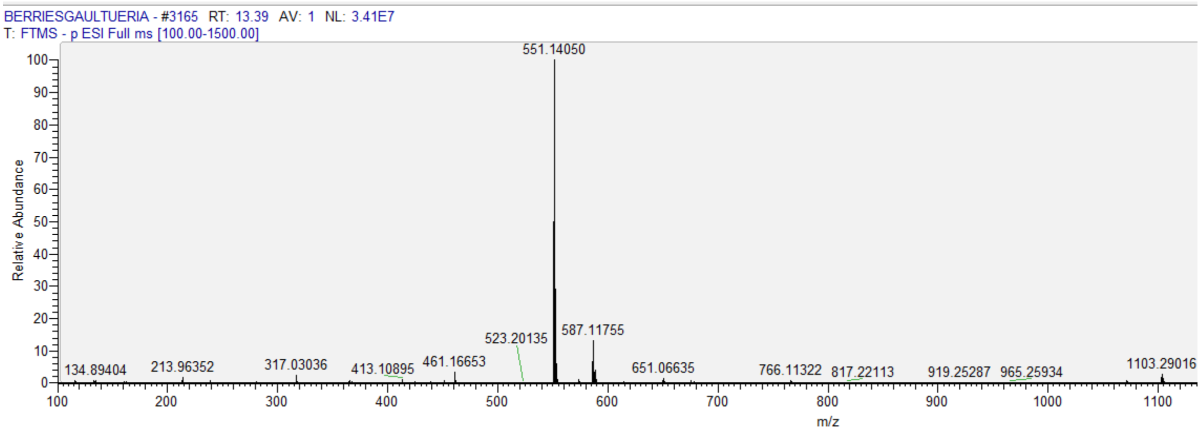

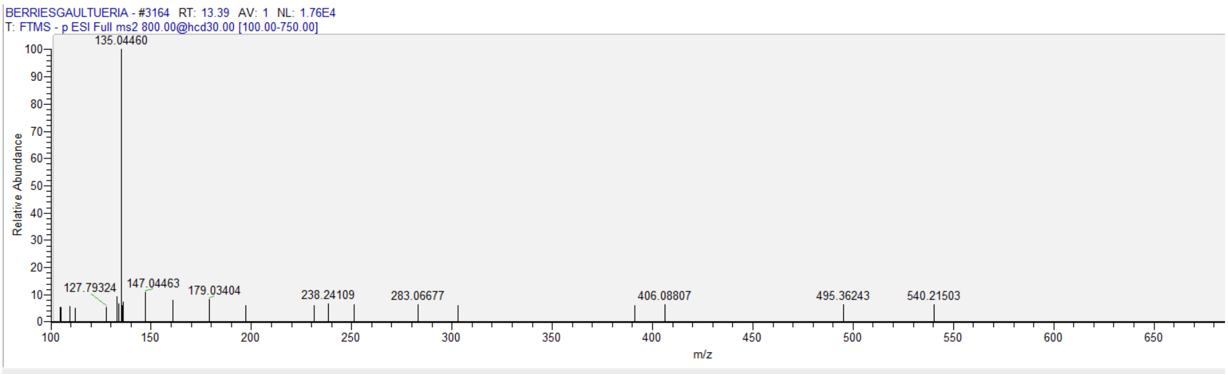

MS and UV absorption at 280 of Myricetin O-rhamnoside m/z = 463.08838 (Peak 14).

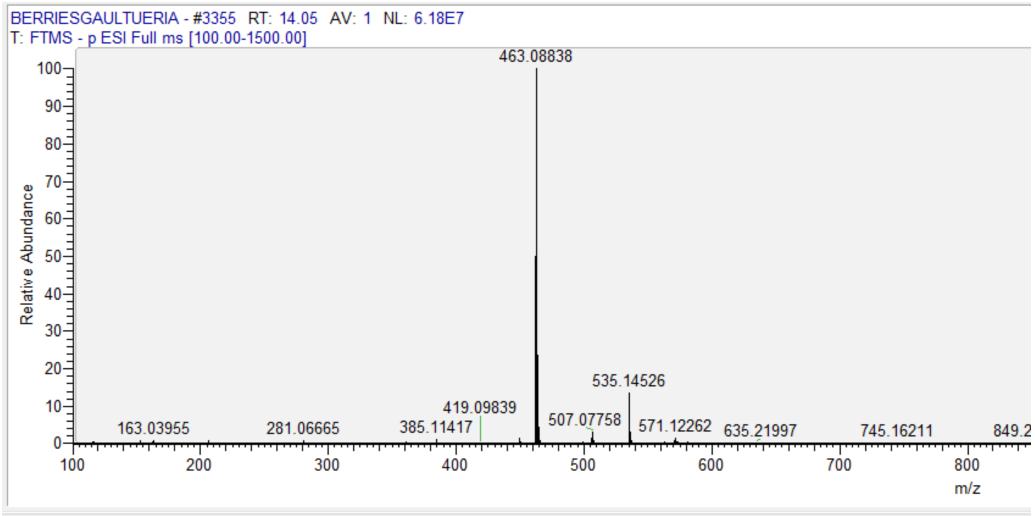

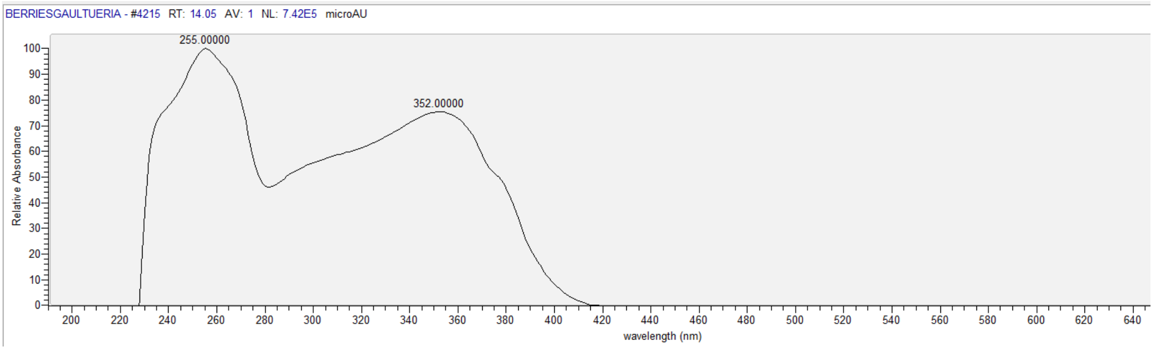

MS and MS² chromatogram of Galloyl derivative *m/z* = 461.07297 (Peak 20).

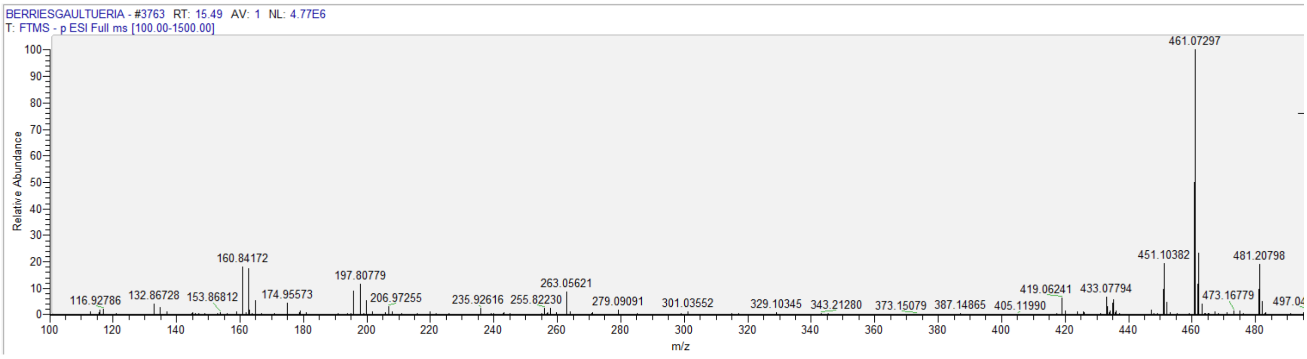

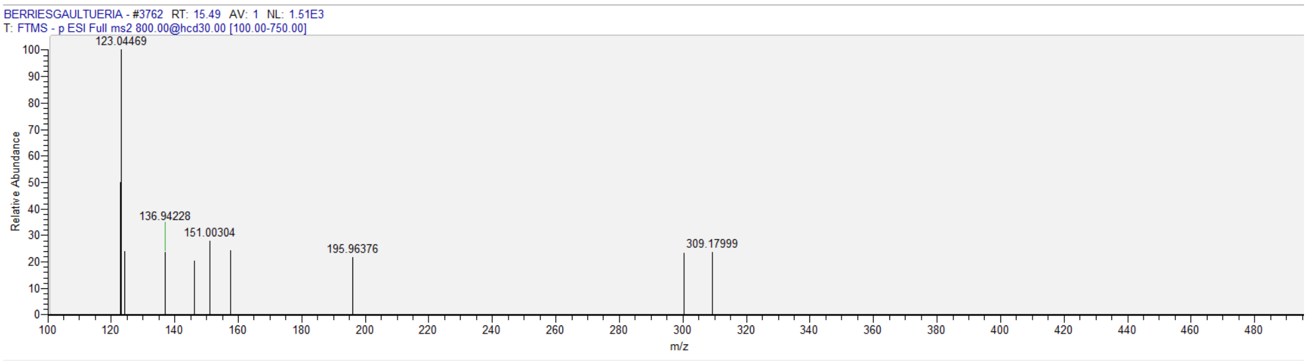

Docking assays into the corresponding catalytic sites of acetylcholinesterase, butyrylcholinesterase, and tyrosinase

Matherial and methods

Enzyme optimizations were carried out using the Protein Preparation Wizard available in Maestro software, where water molecules and ligands of the crystallographic protein active sites were removed. In the same way, all polar hydrogen atoms at pH = 7.4 were added. Appropriate ionization states for acid and basic amino acid residues were considered. The OPLS3e force field was used to minimize protein energy as well. The enclosing box size was set to a cube with sides of 26 Å length.

The centroid of selected residue were chosen based on the putative catalytic site of each enzyme, considering their known catalytic amino acids: Ser200 for *Tc*AChE [1,2], Ser198 for *h*BuChE [3] and His263 for tyrosinase [4]. The Glide Induced Fit Docking protocol has been used for the final couplings. Compounds were punctuated by the Glide scoring function in the extra-precision mode (Glide XP; Schrödinger, LLC) and were filtered on the basis of the best scores and best RMS values (less than 1 unit as a cutting criterion), in order to obtain the potential intermolecular interactions between compounds and the enzymes, as well as the binding mode and docking descriptors. The different complexes were visualised in a Visual Molecular Dynamics program (VMD) and Pymol.

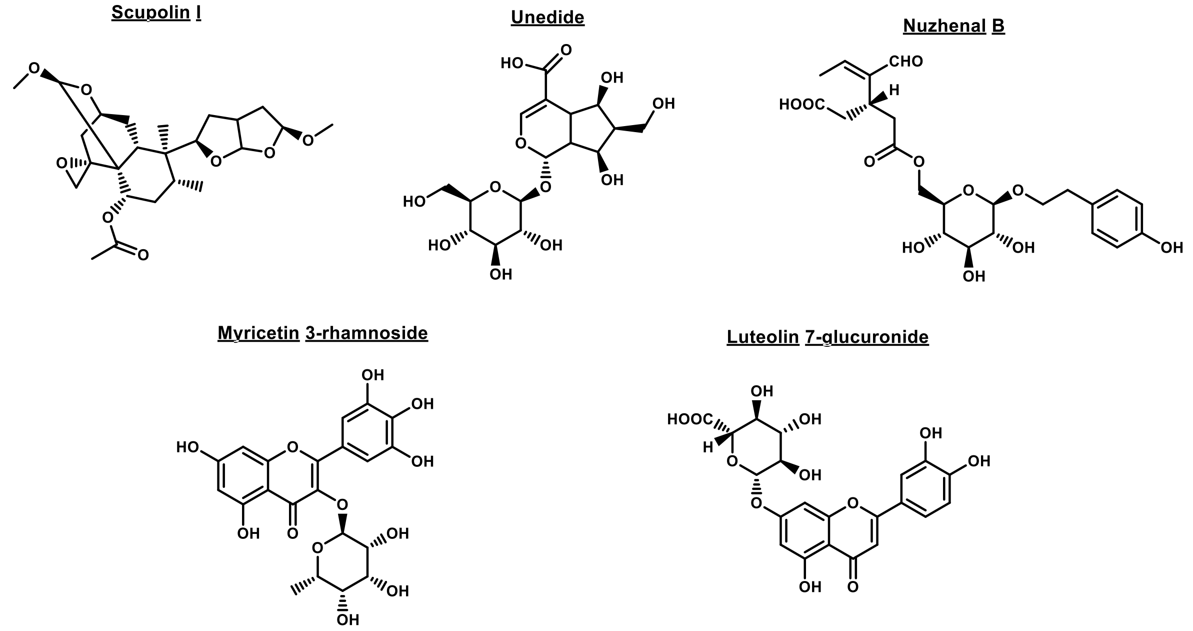

Figure S2. Compounds subjected to docking assays into the corresponding catalytic sites of acetylcholinesterase, butyrylcholinesterase, and tyrosinase.

Results

Figure S3 shows the main interactions in a two-dimensional diagram of the compound with the best binding energy profile of all derivatives found in high proportion within the *Gaultheria pumila* berries extract, and consequently the one that would contribute the most to the inhibitory activity. Each better compound, according to docking experiments, are shown into the catalytic sites of acetylcholinesterase, butyrylcholinesterase and tyrosinase enzymes respectively.

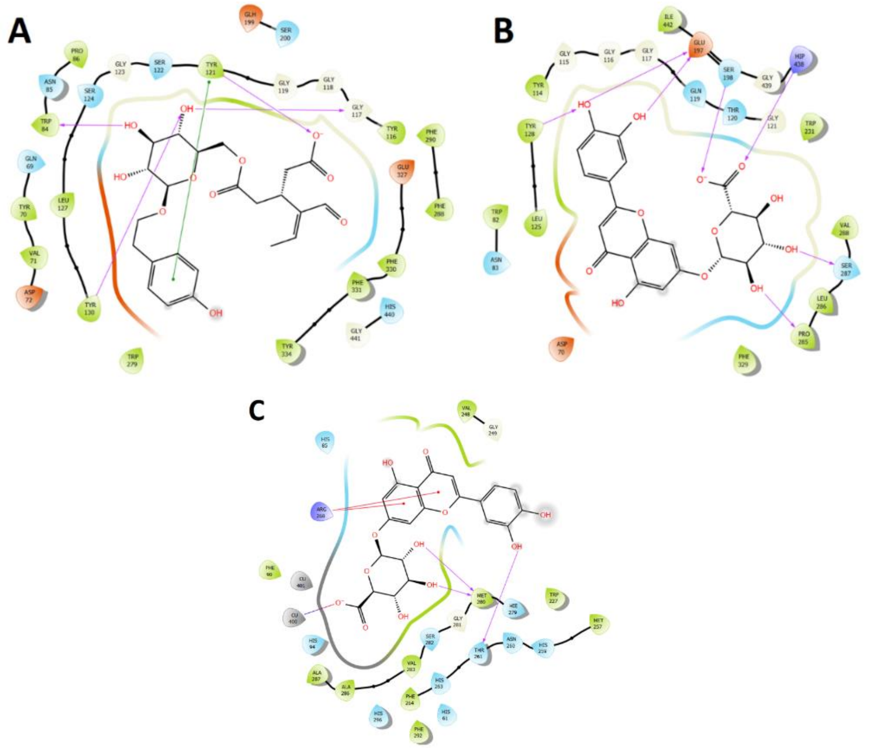

Figure S3. Two-dimensional diagram of (**A**) Most active compound Nuzhenal B (related to docking binding energy) and its main interactions into the acetylcholinesterase (*Tc*AChE) catalytic site. (**B**) Most active compound Luteolin 7-glucuronide (related to docking binding energy) and its main interactions into the butyrylcholinesterase (*h*BuChE) catalytic site.

(**C**) Most active compound Luteolin 7-glucuronide (related to docking binding energy) and its main interactions into the tyrosinase catalytic site.

References

1. Silman, I. Three-dimensional structures of acetylcholinesterase and of its complexes with anticholinesterase agents. *Portl. Press Ltd. 1994 SRC*.
2. Sussman, J.L.; Harel, M.; Frolow, F.; Oefner, C.; Goldman, A.; Toker, L.; Silman, I. Atomic structure of acetylcholinesterase from Torpedo californica: a prototypic acetylcholine-binding protein. *Science 253*, 872–879.
3. Tallini, L.; Bastida, J.; Cortes, N.; Osorio, E.; Theoduloz, C.; Schmeda-Hirschmann, G. Cholinesterase Inhibition Activity, Alkaloid Profiling and Molecular Docking of Chilean Rhodophiala (Amaryllidaceae). *Molecules* **2018**, *23*, 1532.
4. Chen, J.; Ye, Y.; Ran, M.; Li, Q.; Ruan, Z.; Jin, N. Inhibition of Tyrosinase by Mercury Chloride: Spectroscopic and Docking Studies. *Front. Pharmacol.* **2020**, *11*.
